# Finnish inventory data of underwater marine biodiversity

**DOI:** 10.1038/s41597-024-04092-4

**Published:** 2024-11-27

**Authors:** Louise Forsblom, Elina A. Virtanen, Heidi Arponen, Rasmus Boman, Jaakko Haapamäki, Joonas Hoikkala, Niko Kallio, Ville-Juhani Karvinen, Anu Kaskela, Essi Keskinen, Lauri Kuismanen, Lasse Kurvinen, Ari O. Laine, Maiju Lanki, Eveliina Lampinen, Juho Lappalainen, Pekka Lehtonen, Aija Nieminen, Kevin O’Brien, Anu Riihimäki, Henna Rinne, Sonja Salovius-Lauren, Antti Takolander, Karl Weckström, Markku Viitasalo

**Affiliations:** 1 Finnish Environment Institute (Syke), Helsinki, Finland; 2 Finnish Natural History Museum (LUOMUS), University of Helsinki, Helsinki, Finland; 3 Metsähallitus Parks & Wildlife Finland, Helsinki, Finland; 4 Geological Survey of Finland (GTK), Espoo, Finland; 5 North Ostrobothnia Centre for Economic Development, Transport and the Environment, Oulu, Finland; 6 Ministry of the Environment, Helsinki, Finland; 7 Åbo Akademi University, Turku, Finland

**Keywords:** Biodiversity, Marine biology, Environmental impact

## Abstract

Since 2004, marine biodiversity inventory data have been systematically collected with diving, video, and benthic sampling methods in Finland. To date, this collection of data consists of more than 194 000 spatially explicit observations, covering more than 280 aquatic genera, representing mainly macroalgae, vascular plants, water mosses, and invertebrates. We describe the data collection and storage methods, data extraction from national databases, and provide potential users a curated, open-access version of the inventory data. Additionally, examples of data applications and discussion on potential limitations are provided. This extensive dataset can be used in ecological and biogeographical studies to provide general descriptions of biodiversity patterns and species distributions, as well as in more applied studies to support marine management, conservation, and sustainable use of marine areas. The sampling strategy and high spatial and taxonomic resolution allow for statistical modelling, which further increases the usability of the data in research, for instance in identifying key biodiversity areas, estimating biodiversity loss, and assessing efficiency of conservation.

## Background & Summary

Marine biodiversity loss, caused by climate change, habitat degradation, overexploitation, invasive species, and pollution is a global concern^1–3^. These pressures have resulted in considerable decline of species populations, with profound implications for ecosystem stability, food security and human well-being^4–6^. Together with climate change mitigation, various conservation actions, such as establishment of marine protected areas^7^, and other area-based conservation measures^8^, promotion of sustainable resource use^9^, reduction of pollution, and restoration of degraded habitats, are needed to halt the ongoing marine biodiversity crisis^7^. Ecosystem-based marine spatial planning can also alleviate and limit potential negative impacts of human activities on valuable habitats, and on rare, threatened, or functionally important species^10–12^. Effective conservation actions require detailed, high-quality data. Spatially explicit information on where species occur and how their distributions vary across space and time, are crucial, and provide the backbone for conservation and ecosystem-based spatial planning processes.

In Finland, the increasing use of marine areas, and the lack of marine biodiversity information to support marine conservation and the implementation of EU directives (e.g. Habitats Directive (92/43/EEC)), led to the establishment of the Finnish Inventory Programme for Underwater Marine Diversity (Velmu programme, according to its Finnish acronym) in 2004. The primary aim of the inventory programme has been to collect information that can be used to support marine management, conservation, and marine spatial planning.

Since its beginning, the Velmu programme has collected high-quality, spatially explicit information on species and habitats, together with geological and environmental information from the Finnish sea areas. The programme has been executed in two phases. The first phase (2004–2015) focused on gathering observations across the entire Finnish sea area, targeting relatively shallow, photic, biodiversity-rich areas. Based on the information gained, the first popular-science book describing the marine biodiversity of Finland was published in 2017^13^, and its Swedish translation in 2021^14^. This first phase also produced the Velmu methodology guide^15^, which is regularly updated and has since been used by many research projects, as well as environmental administration and consultants, ensuring that Finnish underwater marine biodiversity data are collected in a standardized and comparable manner. In the second phase (2016–2025), inventories have focused on specific areas, habitats, and species, to fill gaps in knowledge around topical research questions, and to support conservation and management of marine areas, according to national laws and international conventions, such as the CBD Global Biodiversity Framework and the EU Biodiversity Strategy^16,17^.

Here we describe the Finnish underwater biodiversity inventory data, which include data collected in Finland´s coastal waters within the Velmu programme, and in separate projects using similar and comparable methodologies. The data consist of over 194 000 observations on macroalgae, vascular plants, water mosses, invertebrates, and fishes, covering more than 280 genera. We describe how the data were collected, deposited, and how to access the data from national databases. Access to biodiversity data is constantly improving as more data become available in international repositories such as GBIF, but local understanding and descriptions of data are still important to support proper use and interpretation of data. These extensive data have been used in various applications, including describing species and habitat distribution patterns^18–23^, species distribution modelling^24^, applied ecological and geological studies^23,25–32^, indicator development^33^, assessing climate change effects^26,34^, systematic conservation planning^35–37^, ecological impact avoidance^38^, and identifying ecologically important areas^30,39–41^. The data have been, and are continuously used by environmental administration in practical work, such as national conservation status assessments^42,43^, management of marine protected areas, planning of measures to mitigate impacts of activities within marine protected areas (e.g. anchoring), and environmental impact assessments^44^. We provide a curated, open-access version of the data to support research initiatives at regional and global scales, such as design of marine protected areas, blue economy, sustainable use of marine areas, and climate change studies.

## Methods

**Fig. 1:**
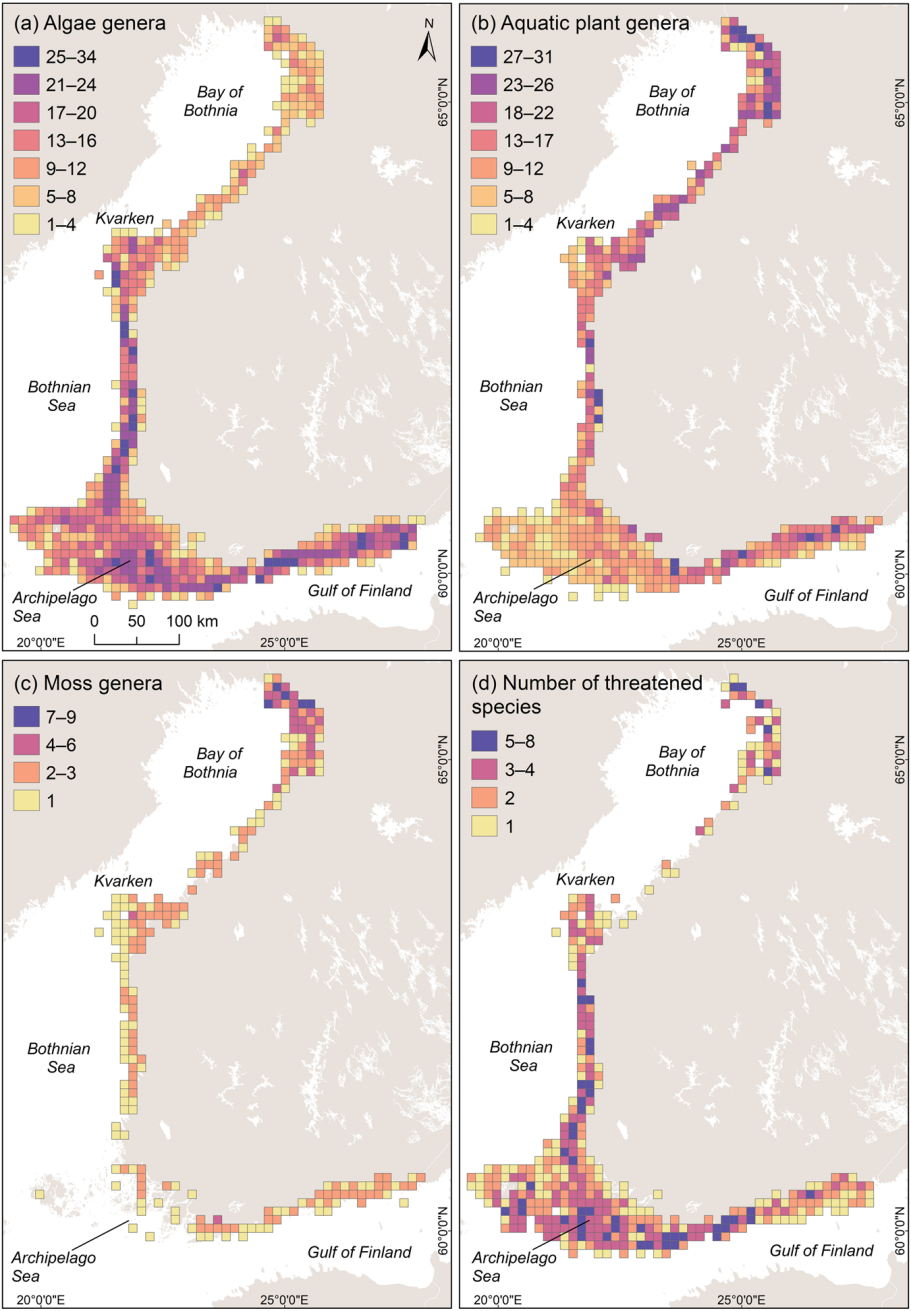
Map shows the number of genera of algae (**a**), aquatic vascular plants (**b**), mosses (**c**), and the number of threatened species (**d**), visualised in 10 × 10 km grids (Jenks natural breaks).

The Finnish territorial waters and exclusive economic zone together cover roughly 21% (81 500 km^2^) of the Baltic Sea. In general, the Baltic Sea is a geologically young, semi-enclosed, non-tidal brackish water sea characterized by a low number of species of either marine or freshwater origin^45^. The marine areas of Finland are characterized by steep environmental gradients in salinity, turbidity, exposure, and geomorphology. These abiotic factors create habitats for a limited variety of species that are highly adapted to these conditions. In the north, the Bothnian Bay is shallow and low in salinity, with exposed shores and a monotonous geomorphology. The Kvarken, located between the Bay of Bothnia and the Bothnian Sea (Fig. 1), acts as a biogeographical barrier between the north and south, beyond which many marine species cannot survive. South of the Bothnian Sea, the Archipelago Sea, with its 52 500 islands, forms one of the most complex archipelago systems in the world^45^. Moving east along the Gulf of Finland, species communities change due to declining salinity: marine species gradually disappear, and freshwater species become more dominant (Fig. 1).

Surface salinity ranges from 7–8 PSU in the southwestern outer archipelago (e.g., Sea of Åland) to nearly zero in the northernmost Bothnian Bay, and to less than 5 PSU in the eastern Finnish territorial waters of the Gulf of Finland. In addition, there is a marked salinity gradient decreasing from the open sea to the innermost archipelago waters and estuaries. Turbidity also follows an inner-outer archipelago gradient, where high concentrations of dissolved and particulate organic matter from rivers, and high on-site primary productivity, elevate water turbidity especially in inner archipelagos. The southern marine areas, especially the Archipelago Sea and the Gulf of Finland, are burdened with various pressures, including eutrophication and hypoxia^46,47^. Finnish marine areas are also extremely shallow, averaging 50 meters in depth, with the deepest point at 293 meters in the Sea of Åland^48^. The cold climate and harsh winters, including ice cover, restrict both species diversity and the possibility to do inventories. The short growing season (which also varies between the north and south), together with fragmented archipelagos, challenge sampling efforts. In addition to perennial macroalgae, some annual species exhibit rapid successional development, with various dominant species appearing later in the summer, whereas some species are only present in the community for a short period annually^49,50^. Poor water clarity in the south and the dark colour of water in the north also mean that the photic zone in Finland is narrow, in some areas only a few meters^19,24^. All these factors need to be carefully considered when choosing methods and developing cost-effective sampling campaigns.

**Fig. 2:**
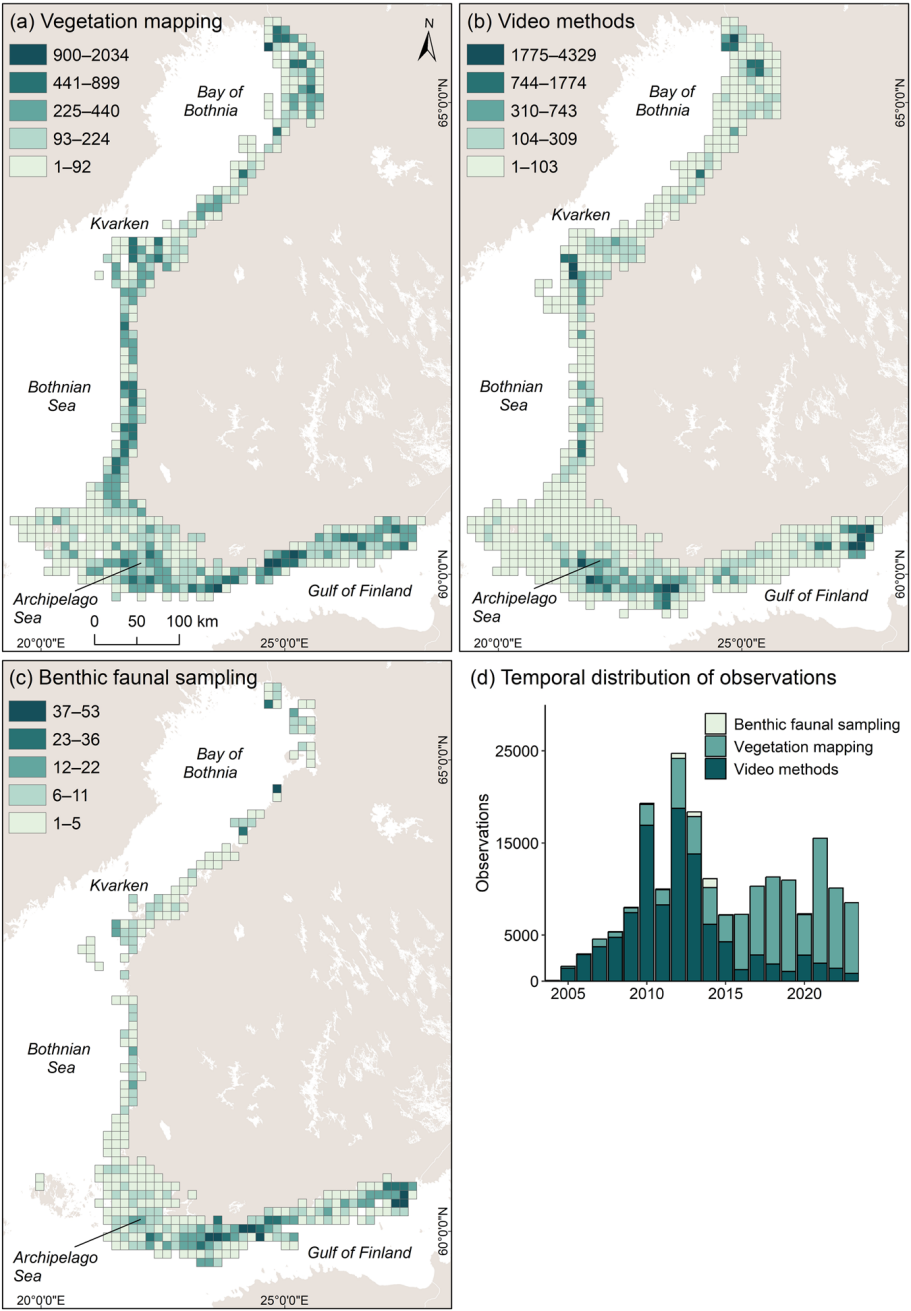
Maps of observations collected per grid cell with vegetation mapping, representing mainly diving and comparable methods (**a**), video observation methods (**b**), and sampling of benthic fauna (**c**), visualized in 10 × 10 km grids (Jenks natural breaks). Observations over the years using the three methods (**d**).

Biological data have been gathered using a variety of methods depending on the aim of the data collection, available resources, and local conditions such as depth, seafloor substrate, and visibility. Almost all data, 97%, have been collected during the growing season from June to September. The collected data can be divided into three main data types based on the sampling approach: (1) data collected by methods that allow for exact identifications to the species level, such as scuba diving, aquascope, and rakes; these methods enable sampling for further species identification, if *in situ* identification is not possible; (2) data collected using video approaches, which offer a good understanding of habitats, but exact species identification is not always possible; and (3) data collected by sampling benthic fauna using samplers for communities on both soft and hard substrates, which subsequently require microscopic analysis (Fig. 2).

The collected data are entered onto standardised spreadsheets and submitted into one of the following databases: (1) LajiGIS: a national database managed by Parks & Wildlife Finland, restricted to the use of environmental administration; (2) POHJE: a national Darwin Core-compliant data interface for benthic fauna observations, which can be freely accessed by making an OData request (http://rajapinnat.ymparisto.fi/api/pohjaelainrajapinta/1.0/); and (3) Åbo Akademi University managed locally for specific uses^37,51–53^. Data from both LajiGIS and POHJE are additionally accessible through the Finnish Biodiversity Information Facility (FinBIF, https://laji.fi/en)^54^. The latter currently has more limited complementary data such as method descriptions and different record identifiers compared to what is presented here. In addition to biological data, other information is also recorded from the sampling site, including depth, the type and percentage cover of the seafloor substrate and amount of loose surface sediment. The substrate classes include bedrock, boulders and stones of varying size, and finer sediments, such as sand or clay.

### Sampling design

Finnish marine areas, especially nearshore areas, are often characterized by archipelagos that create mosaic-like landscapes, making sampling design challenging. Seabed substrates and topography vary over short distances; for instance, the Archipelago Sea represents one of the most geologically variable environments of the Baltic Sea^25,55^. This high variation has significantly influenced the sampling design. Data have been collected either through random stratified sampling or targeted sampling campaigns with specific goals, such as detailed inventories of specific habitats (e.g. reefs or lagoons), threatened and rare species, or by sampling grids of different sizes and resolutions. In the beginning of the Velmu programme, a grid-based sampling design was used within marine protected areas to gain more detailed information on habitat occurrence. Additionally, a random stratified sampling protocol was designed to achieve extensive spatial coverage of data from the entire Finnish sea area and to produce data suitable for species distribution modelling. The stratification was based on depth, water turbidity, salinity, and coarse substrate data, as these factors were considered the most influential affecting species distribution in the northern Baltic Sea^35^. When specific habitats and threatened species were later targeted, habitat^18^ and species distribution models^32^ were used to guide inventories. The following sections provide a general description of all biological inventory methods, and more details are available in the Velmu methodology guide^56^.

### Vegetation mapping

**Fig. 3:**
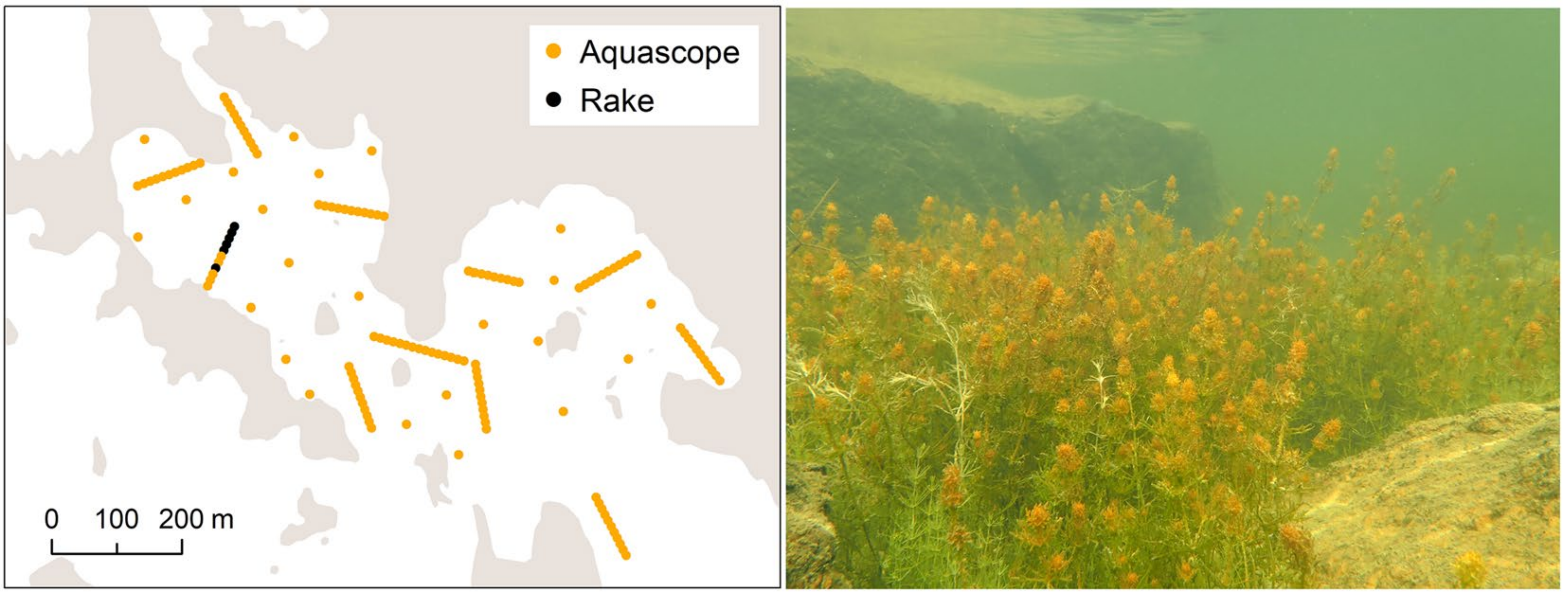
Example map (left) of a lagoon in Replot island in the Kvarken, narrowest part of the Gulf of Bothnia in the northern Baltic Sea, where vegetation mapping was carried out with an aquascope and a rake in summer 2023. The lagoon was in pristine condition, and charophytes, such as Chara tomentosa, were abundant (right). (Photo: Rupert Simon, Parks & Wildlife Finland).

At shallow depths, mainly less than 20 meters, biological data have been collected by scuba diving, snorkelling, or, in the very shallowest waters, wading with an aquascope (Fig. 3). These methods are based on quadrats where the percentage cover is assessed for each macrospecies, and provide detailed information on species, often at high taxonomic level. The dives are generally carried out along a 100 m transect line. Coordinates are recorded at the start and end points of the line and mapped quadrats in between have coordinates calculated based on their position along the transect line. Percentage cover of seabed substrate types is also estimated from the same quadrats, which are chosen at one meter´s depth intervals, every ten horizontal meters, or when the habitat changes. The amount of loose sediment on the seabed, or on top of the vegetation, is also assessed on a scale from 0 to 3. Percentage cover of aquatic plants and macroalgae are assessed from the whole quadrat, and the percent cover of epiphytic algae, i.e., algae growing on other plants, is also evaluated relative to the entire quadrat area. In addition to macrophytes, the method maps the abundance of sessile animals attached to the substratum, estimated either as total percentage cover, in the same manner as macrophytes, or as numbers of individuals.

Inventories using a Luther rake have focused mainly on identifying aquatic plants but have also been used to support or verify video observations, such as identifying water mosses. The Luther rake is useful in turbid waters or areas where drifting plant material limits the use of video and dive methods. Raking is performed multiple times (5–10) at a sampling site, until no new species are observed. The information gained from rakes is usually descriptive and recorded as presence. Not all species are equally well recorded by the rake, as species morphology affects how easily they attach to the rake.

### Video methods

**Fig. 4:**
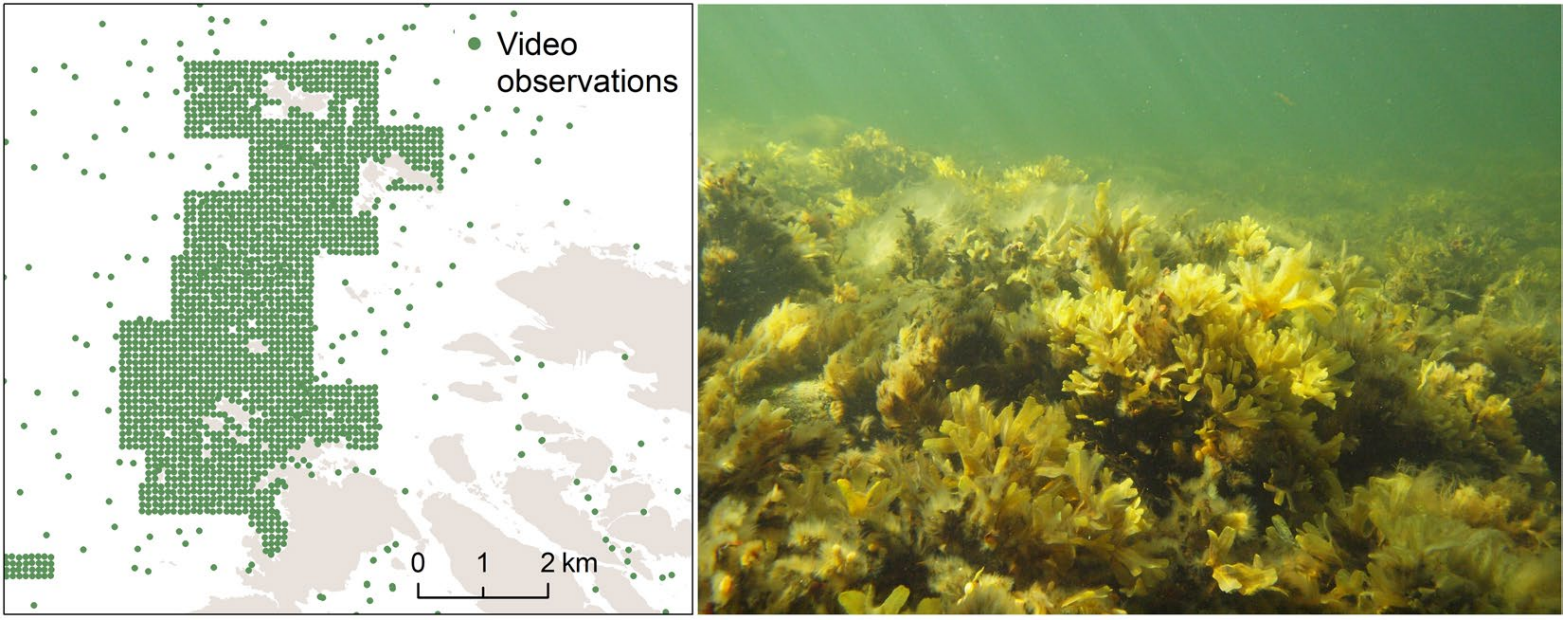
Example map (left) of a video grid in the Bothnian Sea national park, and a species found abundantly there, bladderwrack (Fucus spp) (right). (Photo: Heidi Arponen, Parks & Wildlife Finland).

Underwater video filming has been used for mapping large areas and rapid data collection at the habitat level, such as determining the occurrence of habitat-forming species like bladderwrack (*Fucus vesiculosus*) and blue mussel (*Mytilus trossulus*) (Fig. 4). While video methods are cost-efficient, they are less suitable for species-level identification. Only few large species can be reliably identified from videos (see section Interpretation of videos), and video data collection has been complemented with rakes. Comprehensive, species-level biological inventories need to be carried out by diving.

#### Drop-video

High quality video is necessary for successful interpretation, and data collection is usually done in clear and calm conditions, with a camera system that illuminates the seafloor. During data collection, the video platform is lowered from the boat, and the seafloor is filmed for approximately one minute. The recording starts when the seafloor comes into view and the start and end coordinates, and depth are recorded. To support the quantification of substrate type and presence of loose sediment, the camera is allowed to gently touch the seabed.

#### Remotely Operated underwater Vehicle (ROV)

The ROV unit is a submersible vehicle that incorporates a camera that can record video along transects or circuits lasting from 10 to 30 minutes. It is manually controlled from the surface using a controller and a surface monitor. Videos are shot close enough to the bottom and at a sufficiently slow pace to make subsequent video analysis as easy as possible. The ROVs used for the inventories have been equipped with a positioning system that provides continuous coordinate information along the driven route, which can afterwards be linked to the video footage, creating a position for each film sequence.

#### Interpretation of Videos

The video footage is interpreted to identify habitats, and when possible, species. For the drop-video, the abundance of individuals or groups of organisms is estimated as a percentage cover over the whole minute. For the ROV videos the analysis is performed each time there is a significant change in depth, species coverage or substrate, and these points along the ROV route are each assigned coordinates from the ROV’s positioning data. The coverage of sedentary or sessile animals such as bivalves, sponges, hydroids, cirripeds and bryozoans, are estimated either as percent coverage or as the number of individuals. The seafloor substrate is also assessed as percentage cover. Species identification from video footage is challenging since many species can only be identified by a closer visual inspection or by microscope, and because species may remain undetected, under and amongst the larger canopy-forming species, like bladderwrack. Identification of some larger species is possible, such as eelgrass (*Zostera marina*) or perfoliate pondweed (*Potamogeton perfoliatus*). For species that cannot be identified and attributed a scientific name, descriptive meta-levels are used instead of taxonomic terms, such as *filamentous brown* or *filamentous red* algae or combined groups such as *Pylaiella littoralis / Ectocarpus siliculosus*. As the video image is in constant motion, some species or species group(s) may only appear in the video momentarily. In such situations, only species presence is recorded, and cover is not estimated. Video analyses can also be supported by other data collection methods, such as using a bottom grab or a rake. In such cases, species observations can be included in the video analysis, and recorded as present.

### Benthic faunal sampling

**Fig. 5:**
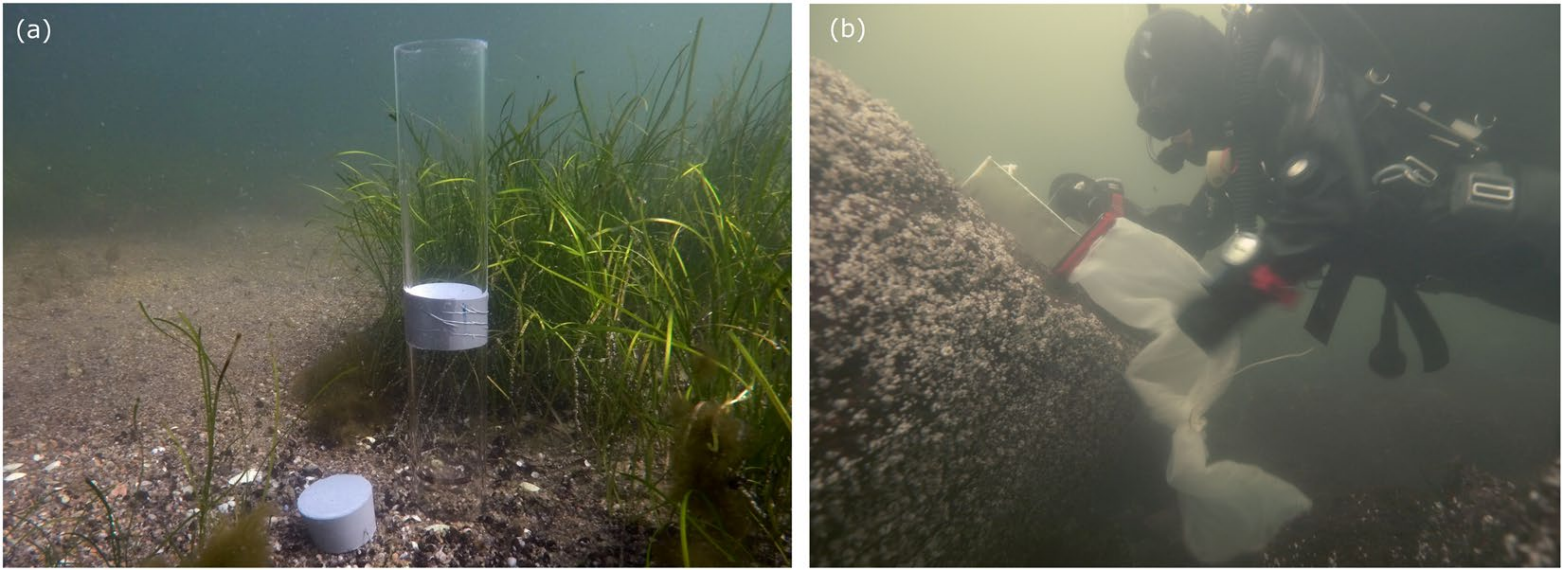
Photos of tube corer (left) and diver taking a Kautsky sample (right). (Photos: Kevin O’Brien and Ari Laine, Parks & Wildlife Finland).

Benthic sessile reef fauna can to some extent be observed using the vegetation and video mapping methods. In- and epifauna require a sampler (Fig. 5), as other methods will mostly miss recording presences of benthic fauna. Data collection starts by choosing a sampler suitable for the substrate; grab samplers on soft bottoms, tube corer on sand, or scuba diver scraping off all flora and fauna within a metal frame on hard bottoms and collecting algal material in net bags. Samples are generally sieved through 0.5 mm, and sometimes, through 1 mm mesh, and remaining species are identified under a stereo microscope. Benthic macrofauna have been identified to species level whenever possible, but genus level or higher taxonomic level has been used if the individuals are difficult or too time consuming to identify (e.g., oligochaetes and chironomid larvae).

Sediment associated fauna is sampled by grab samplers operated from boats or by tube corers operated by divers. The van Veen grab sampler is a reliable tool for collecting sediment samples, covering approximately 0.1 m² of surface area from soft, sandy, or fine gravel bottoms. The Ekman and Petit Ponar grab samplers have much lower sampling area of about 0.02 m^2^. Species with very low densities are less well represented in smaller grab samples, compared to the larger samples taken by the van Veen sampler. Depending on the aim of the sampling, such as the density and distribution of the target fauna, multiple replicates can be collected to increase the representativity of the sample. Tube corers are used to sample communities associated with sandy and gravelly substrates. The Tube corer consists of a plastic tube covering 38 cm^2^. The tube is operated by a diver and pushed to a depth of circa 10 cm.

Since the use of many types of boat-deployed grab samplers is challenging on hard substrates, sampling in shallow areas is usually carried out by diving. Kautsky squares are used to quantify the entire biota of a selected surface area^57^, where species composition and number of individuals, or if possible, species biomass, can be determined. Samples generally represent a discrete habitat zone, such as bladderwrack or red algae belts. The standard sample area is 20 × 20 cm, and the sampler consists of a frame and a collection bag, with a mesh size of 0.3 mm. All living organisms are scraped off the rock into the collection bag using a spatula. In addition to the sampling of hard substrate, epifaunal community data have also been gathered using mesh bags to sample *Fucus vesiculosus* and its associated communities^31^.

### Compiling the datasets

In the following sections we describe how the data files were extracted and curated from LajiGIS and POHJE with additional unique data from the Åland Islands contributed by Åbo Akademi University^37,51–53^. We requested all marine observations from the LajiGIS database^58^ in February 2024 and observations from restricted areas were removed^59,60^. The species observations from LajiGIS are also transferred to FinBIF, but it is not possible to query the data in the exact same format as we are presenting here. Access to the LajiGIS database is restricted to Finnish environmental administration and FinBIF is the official way to access the data. The data include percentage cover data when available and otherwise information on presence. Generally, if a species has not been observed it can be considered to be absent, but the level of detection varies depending on taxa and the method used (see Usage Notes for further discussion). Observations of threatened species *Chara connivens* and *Chara horrida* for the Åland Islands’ sea areas were removed, as this information is considered regionally sensitive. The data in POHJE^61^ is openly accessible and the data shown here is an example extracted using an SQL-query from the internal database. Detailed description of the data and further variables can also be found there. Here we only included data collected within the Velmu programme and associated projects including FINMARINET, NANNUT, TOPCONS.

**Fig. 6:**
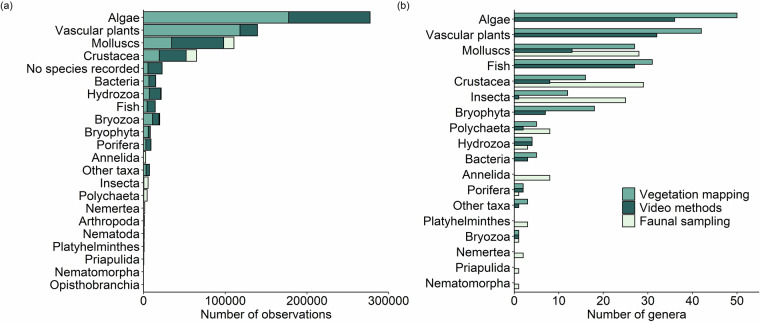
Number of observations (**a**) and number of genera (**b**) per organism group per sampling method, including aquatic vegetation mapping, video- and faunal sampling methods. The group of ‘Other taxa’ indicates observations not targeted by the inventories, or low taxonomic accuracy.

To give an overview of the data and to make it easier to use for research, we provide a species table^62^ with suggestions on how to group the data for different purposes. The table gives an overview of all observed taxa together with their frequency in the data, which helps planning for studies and analyses. The table also contains grouping terms that makes it easier to filter out specific selected groups. The table includes a list of 205 recommended taxa (species or genus) to be used for producing species richness maps for underwater aquatic vegetation using data collected with the vegetation mapping methods. Some of the species included occur on shore in the water line, but were still considered here as the water level in the Bothnian Bay is variable. However, when calculating maximum number of species for the whole area species level should be considered. To facilitate easier use, the species data were grouped into categories: algae, vascular plants, molluscs, crustaceans, hydrozoan, bryozoa, bacteria, fish, bryophyta, porifera, polychaetes, insects, and other taxa (Fig. 6). The group of ‘Other taxa’ includes observations of species not targeted by the inventories (e.g., *Aurelia aurita*), or low taxonomic accuracy, such as observations identified only as macrophytes.

## Data Records

The version of the dataset presented here is available at Zenodo 10.5281/zenodo.13822190^62^. The data consists of three files, containing the vegetation mapping data, benthic faunal data, and a summary table of the species (LajiGIS_AA_vegetation_mapping_video_observations.csv, POHJE_benthic_faunal_sampling_observations.csv, and observation_summary_table.csv, respectively). All datasets are provided as csv files. The file with observations from mapping and video methods include record id, coordinates, sampling method, sampling design, collection date, taxonomic unit, cover, presence absence information, amount of loose surface sediment, grouping variable for taxa, project, and data source (Table 1). Depth and substrate information are not included due to national restrictions. The file with observations from benthic faunal sampling methods include station and sampling id, coordinates, sampling method and associated information, collection date, taxonomic unit, presence absence information, grouping variable for taxa, project, and data source (Table 2). The final csv-file contains summarising information on the observed species and the content is described in Table 3.

**Table 1:** Descriptions of the variables included in the benthic visual observation data.

Column	Description
RecordID	LajiGIS record ID or Åland ID number
intNorthFIN, intEastFIN	Coordinates in EPSG 3067
Method	Sampling method used, including the methods transect, dive, rake, aquascope, video, ROV, and video and rake combined (video + rake), as well as the category other, which includes observations with a 0.25 m^2^ quadrat.
transectID	Used to classify dive transects. NA indicates that the observation is not considered part of a transect.
MethodCategory	Indicates the method category as presented in the present paper as vegetation mapping or by video methods.
SamplingDesign	Numbered from 1–5, 1 = stratified random sampling, 2 = grid 100 m, 3 = grid 50 m, 4 = grid other, 5 = other than 1–4. NA values mean that sampling design is not reported.
Area	The area of the quadrat used for assessing cover, 4, 1 or 0.25m^2^. 99 indicates other area and NA indicates that the information was not available. Not applicable for video methods.
Date	Date in the format yyyy-mm-dd
scientificName	The species name reported at highest taxonomical precision.
verbatimIdentification	The species name reported at highest taxonomical precision, and when not available the taxonomic representation as it was originally recorded but with translations.
Cover	The estimated cover of the observed taxa. When the taxon is observed but the cover is not quantified the cell is assigned the value 9999. This concerns mainly mobile species such as fish, crustaceans, many molluscs, polychaetes, and insects, but also others. Cover is 0 when no species were recorded (verbatimIdentification = *No species recorded*).
LooseSurfaceSediment	Loose sediment on surfaces indicted using a scale from 0–3, where 0 = no sediment, 1 = little, 2 = moderate and 3 = abundant. NA values indicate that the sediment amount has not been quantified.
Group	Species were assigned to groups
Project	The original name of the project the data were collected in including Biodiversea, CoastNet LIFE, ECOnnect, FINMARINET, Kvarken Flada, Mapping and habitat classification of the underwater environment in Geta and Lumparn reports 155^51^ and 152^52^, Mapping marine Natura 2000 habitats in Åland^53^, NANNUT, Parks and Wildlife Finland, SeaCOMBO, SeaGIS 2.0, SEAmBOTH, SeaMoreEco, TOPCONS, Velmu, ÅlandSeaMap^37^
Source	The source where data are stored and was requested from (either LajiGIS or Åbo Akademi)

**Table 2:** Descriptions of the variables in the benthic sampling data.

Column	Description
benthosStationID	Station ID
samplingID	Sampling event ID
Time	Date and time of sampling (yyyy-mm-dd-hh:mm:ss)
intNorthFIN	Coordinates in EPSG 3067
intEastFIN	Coordinates in EPSG 3067
samplerName	Sampling method including Ekman, Ekman x 5, Kautsky sampler, Pedersen, Petit Ponar, Small Van Veen, Tubecorer, Van Veen and Fucus bag.
samplingArea_cm2	Area of sampler if applicable
sieveSizeInMm	Size of the sieve
scientificName	Name of the observed taxa
lifeStage	Life stage of the organism
replicates	Number of replicates from 0–6
unitName	Either quantitative samples with ind/m² or semiquantitative samples with number of individuals.
meanValue	Mean observed quantity
Group	Species were assigned to groups
MethodCategory	Indicates the method category as presented in the present paper as benthic faunal sampling.
Data source	The source of the data

**Table 3:** Summary table of observed taxa, with genus and species grouping, presented together with number of observations, and suggestions on how to filter species for calculating alpha diversity of underwater vegetation.

Column	Description
scientificName	The highest level of taxonomic accuracy observed
verbatimIdentification	The species name reported at highest taxonomical precision, and when not available the taxonomic representation as it was originally recorded but with translations.
Genus	Species genus when applicable
Species_URI	Species Uniform Resource Identifier originating from FinBIF
Group	Unique taxonomic units grouped in the following categories; algae, annelida, arthropoda, bacteria, bryophyta, bryozoa, crustacea, fish, hydrozoan, insecta, marchantiophyta, molluscs, nematoda, nematomorpha, nemertea, opistobranchia, platyhelminthes, polychaeta, porifera, priapulida, unclassified, vascular plants.
IncludeForAquaticVegSpeciesRichness	Expert based suggestion on what to include for calculating alpha diversity for aquatic vegetation. As all species are not identified to species level, this indicates if e.g. genus or other levels of taxonomic precision can be included. Taxonomic units to include indicated by x and taxonomic units that should be merged prior to calculation indicated by the new suggested name. However, when calculating maximum number of species for the whole area species level should be considered.
TerrestrialVegetation	Terrestrial vegetation indicated with x.
N	Number of observations for each taxonomic unit
Source	The source of the data; LajiGIS & ÅA or POHJE
Translation	Translations made to verbatimIdentification

The source of the data is also listed.

## Technical Validation

Data collection requires skilled personnel, and the high quality of the collected data has been ensured by training and cross-comparisons of species identification at the start of each field season. Additionally, the data collector’s ability to assess cover is standardized for both vegetation mapping and video methods. Accurate species identification is demanding, especially since many species may vary in appearance between sea areas. To ensure high quality of data, there have been investigations of the reliability of the data, such as the coordinate accuracy of videos and accuracy of video interpretations^63^. When investigated, cross comparisons on species identifications from videos were generally in agreement between viewers^63^. The creation and upkeep of the Velmu methodology guide has also enabled broad use of consistent methods in multiple projects. In fact, Velmu methods have become a standard, often requested to be used, e.g. by consultants performing similar inventories in Finland. The taxonomic lists are managed by FinBIF^64^, and updated to LajiGIS regularly.

## Usage Notes

Both data handling and methods have evolved during the years and changes are traceable through different versions of the methodology guide^15^. One example of such change in the sampling procedure was the change from substrate-based percentage cover assessment, where the percentage of each species was assessed in relation to each substrate, to cover assessment carried out for the full square. All data were later harmonized to percentage cover, which explains the decimal numbers in older percentage cover estimates. Similarly, the way benthic fauna has been assessed using vegetation mapping and video methods have changed over the years and have later been standardized to percentage cover. We also report mapped sites where no species have been recorded, which allows for further use of data in different type of analyses, such as presence-absence species distribution modelling^32,35^.

The sampling design also impacts data usability. It is important to note that over the years, inventories have concentrated on specific counties and, therefore, sea basins, as the entire coast of Finland cannot be mapped in a single year. Consequently, the spatial distribution of observations varies between years. A (more) conservative approach is to consider a longer time span of multiple years to represent average conditions. If all observations are used for the same analysis, such as for species distribution modelling, it is important to consider how very dense sampling grids in certain areas might affect the results. For example, Virtanen *et al*.^35^ used all observations carried out by diving but included only a random subset of the gridded data, for modelling the distribution of species.

We outline general principles of how to use species data observed from different methods in Table 4. In general, regardless of the method used, considerable issues with incomplete detection remain, especially for mobile invertebrates and fish. These observations should be used with caution, and absences should be considered to contain very high uncertainties, making the data more comparable to presence-only data. In some cases, the data can be considered to present true absences, but in-depth knowledge of species ecology, such as annual succession patterns, is needed. Video methods capture only broad habitats and larger species, and as such, the video data cannot be used to calculate e.g., species richness. The benthic faunal sampling is generally quantitative for species targeted by the sampling and others, such as infrequently observed insects, should be considered with more caution. Concrete examples on how to use the data for e.g. species distribution modelling or to model habitats can be found in Virtanen *et al*.^35^.

**Table 4:** General description of the quality of the data for different taxa using different methods.

Taxa	Quantitative	Presence-absence	Presence only	Notes
Algae	Cover estimates available	Reliably identified to species level using diving, snorkelling and aquascope. Video methods can only be used to identify absences for a selected number of large distinct species.	Rake observations are more accurately used to indicate presence only	Video observations devoid of vegetation can be used to indicate absence
Aquatic plants	Cover estimates available	Reliably identified to species level using diving, snorkelling and aquascope. Video methods can only be used to identify absences for a selected number of large distinct species.	Rake observations are more accurately used to indicate presence only as they don’t capture all species with equal efficiency and some not at all.	Video observations devoid of vegetation can be used to indicate absence.
Terrestrial plants	Cover estimates available		Terrestrial vegetation observed on shore is not observed as consistently as aquatic vegetation. These can be identified from the list described in Table 3.	No systematically collected information available
Mosses	Cover estimates available	Reliably identified to using diving, snorkelling and aquascope. Video methods can only be used to identify absences for a selected number of large distinct species.	Rake observations are more accurately used to indicate presence only	Video observations devoid of vegetation can be used to indicate absence
Sessile fauna	Cover estimates available. Ind/m^2^ from benthic samplers.	Sessile fauna can be reliably identified to species level using Kautsky, diving, snorkelling and aquascope. Using video methods to indicate absence is more prone to uncertainty due to the nature of the method as species can be covered by vegetation		
Epifauna	Semiquantitative; number of individuals from captured with associated to *Fucus* with mesh bags	Fucus bag samples can be used to indicate absences	Epifauna observed using vegetation mapping and video methods should be considered as presence only or at the very least to have imperfect detection.	
Infauna	Ind/m^2^ from benthic samplers.	Benthic grab and core samplers can be used to indicate absences	Infauna observed using vegetation mapping and video methods should be considered as presence only or at the very least to have imperfect detection.	
Fish			Fish observed using vegetation mapping and video methods should be considered as presence only or at the very least to have imperfect detection.	Fish have not been consistently recorded and some projects have not recorded fish at all

## Data Availability

All data processing steps were performed using R version 4.3.0, using packages sf, ggplot2, reshape2, plyr, and readxl. The code used to process original raw data, as well as make all figures is available on Zenodo^62^.
